# Mobile Phone Multilevel and Multimedia Messaging Intervention for Breast Cancer Screening: Pilot Randomized Controlled Trial

**DOI:** 10.2196/mhealth.7091

**Published:** 2017-11-07

**Authors:** Hee Lee, Rahel Ghebre, Chap Le, Yoo Jeong Jang, Monica Sharratt, Douglas Yee

**Affiliations:** ^1^ School of Social Work College of Education and Human Development University of Minnesota, Twin Cities St Paul, MN United States; ^2^ Department of Obstetrics, Gynecology and Women's Health School of Medicine University of Minnesota, Twin Cities Minneapolis, MN United States; ^3^ Division of Biostatistics School of Public Health University of Minnesota, Twin Cities Minneapolis, MN United States; ^4^ Department of Educational Psychology College of Education and Human Development University of Minnesota, Twin Cities Minneapolis, MN United States; ^5^ Division of Hematology, Oncology and Transplantation School of Medicine and Pharmacology University of Minnesota, Twin Cities Minneapolis, MN United States

**Keywords:** breast cancer, mammogram, mobile health, mHealth, mobile app intervention, multimedia text message, tailored message, Korean immigrant women, breast cancer disparity

## Abstract

**Background:**

Despite the increasing breast cancer incidence and mortality rates, Korean American immigrant women have one of the lowest rates of breast cancer screening across racial groups in the United States. Mobile health (mHealth), defined as the delivery of health care information or services through mobile communication devices, has been utilized to successfully improve a variety of health outcomes.

**Objective:**

This study adapted the principles of mHealth to advance breast cancer prevention efforts among Korean American immigrant women, an underserved community.

**Methods:**

Using a randomized controlled trial design, 120 Korean American women aged 40 to 77 years were recruited and randomly assigned to either the mMammogram intervention group (n=60) to receive culturally and personally tailored multilevel and multimedia messages through a mobile phone app along with health navigator services or the usual care control group (n=60) to receive a printed brochure. Outcome measures included knowledge, attitudes, and beliefs about breast cancer screening, readiness for mammography, and mammogram receipt. The feasibility and acceptability of the mMammogram intervention was also assessed.

**Results:**

The intervention group showed significantly greater change on scores of knowledge of breast cancer and screening guidelines (*P*=.01). The intervention group also showed significantly greater readiness for mammography use after the intervention compared with the control group. A significantly higher proportion of women who received the mMammogram intervention (75%, 45/60) completed mammograms by the 6-month follow-up compared with the control group (30%, 18/60; *P*<.001). In addition, the intervention group rated satisfaction with the intervention (*P*=.003), effectiveness of the intervention (*P*<.001), and increase of knowledge on breast cancer and screenings (*P*=.001) significantly higher than the control group.

**Conclusions:**

A mobile phone app–based intervention combined with health navigator service was a feasible, acceptable, and effective intervention mechanism to promote breast cancer screening in Korean American immigrant women. A flexible, easily tailored approach that relies on recent technological advancements can reach underserved and hard-to-recruit populations that bear disproportionate cancer burdens.

**Trial Registration:**

Clinicaltrials.gov NCT01972048; https://clinicaltrials.gov/show/NCT01972048 (Archived by WebCite at https://clinicaltrials.gov/archive/NCT01972048/2013_10_29)

## Introduction

Breast cancer remains the most commonly diagnosed form of cancer in women, with approximately 1 in every 8 women in the United States expected to receive this diagnosis in her lifetime [1]. From 2002 to 2011, the incidence of breast cancer increased significantly by 0.8% annually among Asian American and Pacific Islander women, a sharper rise than among any other racial or ethnic group [2]. The Korean American ethnic group constituted 8.2% of the national Asian American population in 2010, increasing by 38.9% from 2000 to 2010 [3]. Although Korean American women tend to have low rates of breast cancer, breast cancer represents the leading type of cancer in Korean women in the United States [4]. In addition, the incidence in foreign-born Korean women demonstrated the greatest increase among Asian American subgroups, at 4% per year [5] between an initial period from 1988-1992 to a second period from 1997-2002 [6]. Accordingly, breast cancer incidence is approximately two times higher in US Korean women compared with native Koreans [6].

Most concerning, Korean Americans have strikingly low rates of cancer screening, including mammography. Breast cancer screening can reduce mortality by detecting cancers at an earlier stage of disease progression when the likelihood of survival is high. Overall, mortality reductions from the routine use of mammograms have been estimated between 19% and 40% [7]. As mammography has shown to effectively detect signs of breast cancer before they can be seen or felt, the American Cancer Society (ACS) recommends annual breast cancer screening for women in the age group of 45 and 54 years, as well as biannual or annual screening depending on a patient’s risk for women aged 55 years and older [8]. Whereas there is controversy over the screening interval and age to start screening for mammography [9], the recommendation of screening is warranted in a population with a rising incidence.

In a sample from the California Health Interview Survey in 2003, Korean Americans reported the lowest engagement in nearly every type of cancer screening test, with over half (57.4%) of Korean American women indicating that they had never received a mammogram or that their last mammogram had taken place over a year ago [10]. Among diverse Asian American ethnic groups, Koreans have repeatedly demonstrated the lowest rates of up-to-date mammography screening, ranging from 22% to 57% [10-18]. Even more alarming, in one study [17], the proportion of Korean American women aged 40 years and older who had never engaged in mammography screening was estimated at 85% for those aged 40 to 49 years and 71% for those aged 50 years and older—much higher proportions than any of the other Asian ethnic groups surveyed. The rates of mammography among Korean American women across samples fall well below the Healthy People 2020 target of at least 81.1% of women aged 50 to 74 years having received a mammogram within the past 2 years [19].

Numerous barriers to breast cancer screening among Korean American women have been identified through previous research, which can be categorized as related to health care access, immigration history, and culture. Health care access factors include low rates of health insurance coverage [10,11,14,18,20] and lack of a usual source of care [10,13,14]. As many Korean Americans are foreign born, their recent immigration status may inhibit screening behaviors, in part, because of limited English proficiency [10,11,20-22]. Attitudes influenced by culture present barriers to screening as well. Some Korean women believe there is no risk of getting breast cancer [22], especially if one eats a healthy diet, has no family history of cancer, does not think or worry about it, and has not had multiple sexual partners or abortions [23]. Furthermore, some beliefs include that Korean women only get breast cancer if they work outside the home and do not have time to breastfeed their children [21] or that the development of cancer depends solely on fate [23]. Perceptions of the purpose of seeing a health care provider may also influence screening behavior, including that receiving a mammogram is embarrassing [16] and that it is only necessary to visit a health care provider when ill [16,24]. Older Korean immigrant women have expressed significantly different health beliefs pertaining to breast cancer screening than their younger counterparts [25]. Finally, health literacy, especially knowledge about mammography, strongly impacts Korean American women’s screening behaviors [12,20,22,26,27]. In one study, knowledge of screening guidelines emerged as the single most important predictor of regular mammography, with greater knowledge increasing the likelihood of mammography by over 10 times [22]. Many of these barriers are modifiable, including health literacy, health care system factors, and cultural barriers, signaling targets for cancer screening promotion interventions. Factors that facilitate uptake of breast cancer screening serve as valuable targets as well. Among Korean American women, these facilitators include higher perceived benefits [15,26], more confidence in screening techniques [26], greater perceived susceptibility to breast cancer [15], and lower perceived barriers [12,26,28,29].

Due to cultural variations among different Asian ethnic groups, there has been a call to develop tailored approaches to reduce barriers and promote screening [17,21,23], yet little intervention development has successfully addressed this issue in Korean American women. Available evidence suggests that although a number of cancer prevention approaches geared toward Korean American women have been introduced, including interactive education sessions [12,27,30], a printed brochure [31], and a community intervention with church-based workshops, and financial incentives [24], these interventions have had limited impact on promoting receipt of breast cancer screening. Key reasons behind such limited success include Korean American women being a hard-to-reach population [24,31] and the lack of tailoring to overcome cultural and personal barriers in previous interventions [24]. Designs that have demonstrated positive results tend to be community focused and provide improved access to preventive health care [32,33]. However, studies reporting effective interventions, such as a Korean-language photonovel [34], have sometimes measured only changes in knowledge of screening guidelines or intention to receive a mammogram rather than the actual receipt [12,35]. Interventions that actually improve mammogram receipt, such as a class combined with lay health worker follow-up counseling and navigation assistance [32], tend to be resource-, labor-, and time-intensive with restricted feasibility for widespread dissemination across the nation. In addition, though reservations about screening vary even within a single ethnic group, past interventions have not personally tailored interventions to each individual’s concerns.

Addressing the gaps from previous research, this project sought to harness mobile phone technology as a means to enhance preventive health care among the Korean American population. Innovative health interventions increasingly incorporate the use of the Internet for a variety of reasons, including low cost and resource needs, convenience, overcoming the isolation of patients, reducing stigma, and allowing greater user control [36]. Mobile health (mHealth), refers to the use of mobile technology for health information delivery or the improvement of health outcomes [37]. In recent years, mHealth has emerged as a direct and effective medium to change health behaviors, demonstrating success in improving weight loss, metabolic control, blood pressure, diabetes management, stress levels, physical activity, asthma symptoms, medication adherence, hemoglobin A1c levels, smoking cessation, and self-efficacy [38-40]. However, there has been criticism that previous mHealth interventions lacked methodological rigor [41], were not driven by established theories [42], and have rarely been customized to meet the needs of unique individuals [37]. This study incorporated individually and culturally tailored messages into an mHealth intervention, evaluated through a randomized controlled trial (RCT). To our best knowledge, to date, a mobile phone app has not been adapted for mammogram promotion. Shaped by the Fogg behavioral model (FBM) [43] and the concept of persuasive technology [44], this study developed the mMammogram app, a mobile phone app–based intervention designed to motivate Korean American women to undergo an annual mammogram. In response to the fact that all seven of the identified previous intervention studies to promote breast cancer screening in Korean Americans utilized quasi-experimental designs [35], this study employed a novel RCT design with a comprehensive approach that addresses individual, cultural, and system barriers. Through the mobile phone medium, the intervention covered broad content areas and specifically tailored messages to overcome known barriers. This study aims to assess the efficacy of the mMammogram intervention combined with health navigator services, which were designed to motivate Korean American women to undergo breast cancer screening, as compared with the control brochure group. The four hypotheses were as follows: compared with the control group participants, the participants who received the mMammogram app intervention (1) would show greater positive change in knowledge, attitudes, and beliefs about breast cancer screening; (2) would demonstrate greater readiness, or intent, for mammography; (3) would report having received a mammogram at a higher rate; and (4) would express greater acceptance of and satisfaction with the intervention. As no previous study has evaluated a mobile phone–based breast cancer screening intervention in this underserved group, our pilot study sought to provide important insights as to the feasibility and acceptability of the mMammogram intervention, with the ultimate objective to reduce breast cancer disparities by enhancing adherence to screening guidelines.

This study applied Fogg Behavioral Model (FBM) [43], which has originated from persuasive technology [44], to overcome attitudinal and behavioral barriers to screening. Persuasive technology refers to a type of computing system intentionally designed to influence individuals to change maladaptive attitudes or behaviors by giving social cues to elicit certain responses from users [43,45]. The principles of FBM, which have become commonly employed in preventive health care, were utilized to increase self-efficacy and steer a process of change. The FBM explains how persuasive technology can provide an effective mechanism for behavioral change; because behavior is a product of motivation, ability, and triggers, a person must be sufficiently motivated, have the requisite ability, and be appropriately prompted to perform a target behavior [43]. All three factors must simultaneously be present for the new behavior to occur, which can be facilitated through technological devices. The FBM guided the design and development of the mMammogram intervention in first identifying barriers, then creating customized motivators, and finally providing timely triggers. In addition, the health belief model (HBM) [46] provided direction on uncovering the factors, such as perceived susceptibility, perceived severity, perceived benefits, perceived barriers, cues to actions, and level of self-efficacy, to target for change for each individual.

## Methods

### Study Design

In this two-arm RCT, participants were enrolled and screened for eligibility and informed consent was obtained. All participants then completed the baseline assessment (pretest) through an in-person interview at her preferred place and time before being randomized into the mMammogram intervention group or the usual care control group. No blinding of participants or study personnel was implemented. Control group participants received a printed brochure written in Korean that informs guidelines for breast cancer screening. For participants assigned to the intervention group, the research team downloaded the mMammogram mobile app onto each individual’s personal mobile phone or a mobile phone lent to the participant by the research team for the duration of the intervention. The intervention period lasted 1 week with a 6-month follow-up. Postintervention assessment interviews that utilized an extended version of the baseline questionnaire with additional questions regarding acceptability of the intervention took place at 1 week and 6 months following intervention completion. The 1-week posttest was conducted in person, whereas the 6-month follow-up test was administered via phone. Questionnaires were first developed in English and then translated into Korean using a back-translation method. All interviews were carried out by trained bilingual interviewers experienced in conducting in-person interviews in the Korean language and certified through intensive training, including review of written interview protocols, critical observations, and mock interviews. The institutional review board of the University of Minnesota approved study procedures.

### Participant Recruitment, Assignment, and Retention

Using a multipronged recruitment strategy, 149 Korean American women were recruited for participation in this RCT. Eligibility criteria included the following: (1) being a Korean American immigrant woman, (2) aged 40 to 79 years, (3) who had not received a mammogram in the past 2 years, (4) lived in Minnesota, (5) possessed an active email account, and (6) were willing to use their own mobile phone or a mobile phone borrowed from the research team for the mobile app intervention. The exclusion criteria included those who (1) were born in the United States or immigrated to the United States as minors (under 18 years), (2) received a mammogram in the past year, and (3) aged under 40 or 80 years and older. Participants were recruited using flyers and brochures in the Korean language that were distributed to churches, temples, clinics, social service agencies, ethnic community centers, beauty salons, and ethnic markets serving the Korean American community. These materials specified the purpose of the project, eligibility criteria, and study personnel contact information. Members of the research team also made presentations at Korean churches and community centers and generated coverage in the Korean American ethnic press.

To obtain an adequate sample size, the project aimed to enroll 150 women with 75 in each arm, assuming an 80% retention rate. It was anticipated that a two-sided two-sample *t* test would be used at the conventional 5% type I error rate and 80% statistical power. A final sample size of 60 in each arm would allow the detection of an effect size of approximately 0.5, a difference in the average score equal to half the standard deviation (SD), conventionally considered a large trial. As previous research enabled an assumption that 20% of the control population would receive mammograms, a group size of 60 allowed the detection of difference in the proportions at 25% using a chi-square test at a 5% type I error rate and 80% statistical power.

After enrolling 149 participants, 144 provided informed consent and completed the pretest. Before the next phase, 13 participants were automatically released from participation after realizing they had received a mammogram in the past 2 years, rendering them ineligible. A total of 131 participants were randomized to the intervention and control groups by an approximately 1:1 ratio (intervention: n=68; control: n=63). The method of sequentially numbered, opaque sealed envelopes (SNOSE) was used for randomization [47]. Sealed identical envelopes were given to participants with a code designating intervention or control group written on a piece of paper on the inside; there were no detectable differences between the envelopes. Over the intervention period, 3 participants dropped out from each group (intervention: 2 loss of contact, 1 cognitive impairment; control: 1 refused, 1 not eligible [remembered receipt of mammogram within past 2 years], and 1 incomplete data). Although 65 participants in the intervention group and 60 participants in the control group completed the intervention period and all measures, 60 participants from each group were analyzed, as 5 participants in the intervention group were dropped from the analyses because of ineligibility. The 5 participants reported that they actually received the mammogram in the past year; the research intervention reminded them of the receipt of the mammogram when watching a video of a mammogram procedure. In sum, among the initially recruited 149 participants, 19 participants were screened out from the study before or after the intervention because of ineligibility. Among the remaining 130 participants, 10 participants left the study, thus yielding a 7.7% attrition rate. Each participant received US $20 for each face-to-face interview, plus US $20 reimbursement for text message data fees over the 6-month period in the intervention group.

### Community Advisory Board

Drawing on a community-based participatory research approach, a community advisory board (CAB) was formed to provide guidance throughout the process of study development, execution, and dissemination of research findings. The CAB consisted of 5 members of the local Korean American community, including representatives from the Korean Service Center; Korean American Association; Korean American Women’s Association in Minnesota, a university student group; and a Korean ethnic church. In bimonthly meetings, members of the CAB provided input in generating the format and content of text and multilevel and multimedia messages, ensuring cultural relevance. The CAB also assisted in devising strategies for participant recruitment and retention, enhancing the accessibility of the website, interpreting preliminary findings, and suggesting approaches for dissemination in the community.

### mMammogram Intervention Development

The process of development for the mobile phone app, mMammogram, involved five main steps: (1) forming a CAB, (2) identifying barriers and mobile phone usage patterns and preferences, (3) creating motivators, (4) tailoring message content, and (5) developing appropriate triggers. After CAB members had been identified, a series of focus groups with Korean American women in their 40s and 50s were conducted to ascertain barriers, motivators, and mobile phone usage patterns. Each session lasted 1.5 to 2 hours, during which participants discussed their current knowledge of breast cancer and screening guidelines; individual, structural, and cultural barriers to screening; current mobile phone usage habits, including text and picture messaging; short message service and multimedia messaging service subscriptions; and ideas regarding the most effective content, type, and frequency of messages to promote screening.

Utilizing data from the focus groups, feedback from the CAB, and input from persuasive technology consultants, the content of the text, multimedia messages, and follow-up schedule were designed and finalized. Special emphasis was given to cultural health beliefs and misconceptions about breast cancer screening, such as the assumption that the absence of symptoms means good health, profound embarrassment related to physical exams, and fatalistic views of cancer. The system was designed to be both personally tailored and interactive, with the content, number, and timing of daily messages adapted to each individual. To keep messaging fresh and nonrepetitive, a database was generated with an ample amount of messages so that the type and content of messages could be varied over the week. The overall computer system for the intervention consisted of five components: (1) a Web-based application to enroll participants, set user preferences, display the global positioning system (GPS) navigation system with area clinic information, and upload text and multimedia messages; (2) a database to store participant records, rules, and messages sent and received; (3) a program to establish the appropriate timing of messages, determine which messages to send, and process received replies; (4) a text-message delivery or reception platform; and (5) a health navigator for assistance navigating cancer screening information, addressing technical problems, and providing transportation and interpretation services. The system also had tools enabling continuous technical monitoring to recognize anomalies that might indicate an individual was having difficulties with the mobile app. In these cases, the health navigator contacted the participant to prevent user frustration and increase adherence and satisfaction.

A series of three usability tests of the mMammogram system prototype were conducted with 5 focus group participants before the RCT, with feedback incorporated into the final mobile app. Each participant was asked to describe her evaluations of the wording of text messages and delivery of accurate information, quality and length of the videos, ease of message delivery, quality of emoticons and the appropriateness of their locations in text message sequences, quantity of interactive messages each day and difficulty responding to each question, overall length of messaging each day, technical problems, and their general impressions of the app for learning about breast cancer screening. On the basis of this feedback, the app was revised. The second and third usability tests were conducted in a similar manner with the same 5 participants, leading to further refinement.

At the outset of the RCT, following initial recruitment, pertinent information about each participant was collected during baseline face-to-face interviews regarding current knowledge of breast cancer, structural or cultural barriers to screening, level of intention to receive a mammogram, and personal preferences around SMS and MMS. In addition, a true or false questionnaire was employed to assess each participant’s personal risk for breast cancer. Participants’ responses in interviews and to the questionnaire were used to tailor messages to each individual.

The actual intervention was delivered in Korean over a 7-day period. Each day we sent 8 to 21 messages to participants over the course of the 7-day intervention. In the last text message of each day, the specially designed mMammogram logo was included to symbolize the conclusion of the intervention for that day. The week-long program allowed sufficient time to highlight various topical areas, including breast cancer, screening guidelines, and types of screening; breast cancer risk factors; individual, structural, and cultural barriers to screening; communication strategies; follow-up for test results; and information on local clinics. Messages followed a trajectory from basic knowledge building to specific strategies aimed to enhance motivation for and access to mammography. Approximately half of the messages requested a reply, providing a balance between education and motivation. An incentive system was employed to increase participant engagement; for each response to a question or a prompt, regardless of whether a participant answered a knowledge question correctly, she could earn a digital pink ribbon and collect these ribbons throughout the intervention period. Recognizing that visual messages can be particularly persuasive, some messages included illustrations, reference photos, and video clips. Video messages featured, for example, Korean American women sharing their personal experiences with mammogram screening, including how they have handled issues related to their cultural beliefs.

To increase accessibility to screening services, a website was created containing a list of area clinics and indicating those that provide free or lost-cost mammograms. All participants received a link to the website that could be accessed by a mobile phone or computer. The list provided information about all clinics, including office hours, types of health insurance accepted, possible free or low-cost options, and physician profiles. In addition, an embedded GPS navigating system allowed participants to determine the distance of clinics from their residence and directions to their chosen clinic. At the end of topic-based message sequences, participants were sent questions as triggers to set up appointments for a mammogram. In order for a trigger to be effective, it had to be noticed, associated with the target behavior, and sent at a suitable time. Participants were sent triggers such as, “Would you like a list of clinics in your area that offer screening?” Those who replied *yes* were sent links to the website with the customized contact information for local clinics and a message with a motivational statement such as, “Call today for an appointment!” A health navigator was available to assist participants in obtaining the necessary resources, appointments, or transportation to receive a mammogram.

### Control Condition

Participants assigned to the control group received usual care that consisted of the mailing of printed materials in the Korean language with contact information of health navigator for questions regarding information we provided in the brochure. This approach has traditionally been used by ethnic health service agencies to promote cancer screening. The materials included a brochure with information on breast cancer and relevant screening guidelines from the ACS, as well as a list of community clinics, indicating those that offer low-cost or free mammography. The control group completed the same assessment schedule (baseline, 1 week post intervention, and monthly follow-up test) with the exclusion of the intervention.

### Measures

Mammogram receipt was the primary outcome measure, whereas breast cancer knowledge, health beliefs, cultural attitudes, level of intention, and participant’s satisfaction and opinion about the effectiveness of the intervention constituted secondary outcome measures. Control variables included sociodemographic characteristics (age; educational attainment; employment, income, and financial status; marital status; family members; and residence); family cancer history; health status; health care access; immigration information; lifestyle variables related to exercise, drinking, and smoking; and past breast cancer screening experiences. Outcome measures were operationalized as follows:

#### Mammography Receipt

Mammography receipt or a scheduled appointment after the intervention was collapsed into one variable and assessed through self-report (yes or no), which has been found to be reliable in cancer screening research [48]. Participants’ mammography receipt was tracked for 6 months after the intervention (up to follow-up period).

#### Breast Cancer Knowledge

Breast cancer knowledge was measured by the breast cancer knowledge test [49], which has been validated with women from diverse cultural groups [50-52]. The test was revised to reflect current ACS breast cancer screening guidelines. The final knowledge scale consisted of 28 true or false items, and the score was computed by the number of items the participant answered correctly. The internal consistency for the present sample was acceptable (alpha=.77 for the pretest, alpha=.75 for the posttest).

#### Health Beliefs

Health beliefs were measured by Champion’s health belief model (HBM) scale [53,54]. Items in the HBM scale map to three main variables used in this study: perceived susceptibility (3 items), perceived benefits (5 items), and perceived barriers (11 items). The scales have demonstrated high reliability and validity in the past, with ethnically diverse sample populations [55-58]. The full list of health beliefs assessed consisted of perceived susceptibility (3 items), perceived benefits (5 items), and barriers to receiving mammogram (16 items), as well as prevention orientation (5 items), self-efficacy of breast cancer screening (8 items), and distrust toward health professionals (5 items). All items were on a 4-point scale ranging from strongly disagree to strongly agree or from unconfident to confident. Higher item scores were indicative of stronger belief for the given construct, and scores of each construct were computed by the sum of item scores. The internal consistencies for the present sample was as follows: perceived susceptibility: alpha=.87 for the pretest, alpha=.73 for the posttest; perceived benefits: alpha=.70 for the pretest, alpha=.75 for the posttest; barriers to receiving mammogram: alpha=.89 for the pretest, alpha=.90 for the posttest; prevention orientation: alpha=.40 for the pretest, alpha=.55 for the posttest; self-efficacy of breast cancer screening: alpha=.89 for the pretest, alpha=.93 for the posttest; and distrust toward health professional: alpha=.72 for the pretest, alpha=.70 for the posttest.

#### Cultural Beliefs and Attitudes

Cultural beliefs and attitudes toward breast cancer screening were captured through 6 items from Tang et al’s inventory [59] of cultural barriers to screening among Asian American women and 3 items regarding fatalism from a questionnaire developed by Taylor et al [60]. Besides fatalism (3 items), other attitudes measured were modesty (5 items), social support (6 items), and fear of discovery (1 item). All items were on a 4-point scale ranging from strongly disagree to strongly agree, with higher item scores indicative of stronger belief for the given construct. Scores of each construct were computed by the sum of item scores. The internal consistencies for the present sample was as follows: modesty: alpha=.70 for the pretest, alpha=.69 for the posttest; and social support: alpha=.57 for the pretest, alpha=.72 for the posttest. The internal consistency for the fear of discovery is not computable because it is a single-item scale.

#### Level of Intention

Level of intention to obtain a mammogram was informed by the stages of change in the transtheoretical model [61], which suggests that people move through a series of progressively more committed stages toward adoption of a new behavior. Adapting the stages of change to intention for mammography, participants were asked to indicate their level of intention to receive a mammography in the future on a 4-point scale (1=not within a year, 2=within a year, 3=within 3 months, and 4=within 1 month). One week after the intervention, the intention was reassessed among participants who had not received a mammography since participation in the study.

#### Participant Satisfaction

Participant satisfaction regarding the intervention they received was assessed using a 4-point scale item ranging from very dissatisfied to very satisfied 1 week after the intervention. In addition to general satisfaction, participants’ willingness to recommend the intervention they received and intention to receive a mammography after this study were also measured using yes-or-no items 1 week after the intervention.

#### Intervention Effectiveness

Intervention effectiveness was measured by a 4-point scale item ranging from very ineffectual to very effectual. In addition to the general effectiveness of the intervention, participants’ perceived level of knowledge about mammography was measured on a 3-point scale item (1=same, 2=improved, and 3=very improved).

### Data Analysis

The data analysis included 60 participants in the intervention (ie, mMammogram app) group and 60 in the control group (ie, brochure) who completed pre- and posttest questionnaires. Before addressing proposed hypotheses, group equivalence in terms of baseline characteristics (ie, sociodemographics, family cancer history, health status, health care access, and past breast cancer screening experiences) was examined using *t* test and chi-square tests. For hypotheses 1 and 2, group equivalence at the pretest was first examined using the two-sample *t* test. Then, group differences in terms of changes in the given constructs were tested using a mixed-effect analysis of variance (ANOVA). The mixed-effect ANOVA includes both within-subject (ie, repeated measures) and between-subject factors (ie, independent variable for which participants are assigned to one of the different conditions) and aims to examine whether there is an interaction between these two factors on the dependent variable. In the context of this study, time (pre- and posttest) represented the within-subject factor, whereas group (app vs brochure) represented the between-subject factor. For hypothesis 3, the percentage of participants from each arm who received mammograms or scheduled an appointment was compared using the chi-square test. Finally, for hypothesis 4 **,** averages of general satisfaction and effectiveness scores from each group were compared using the two-sample *t* test. Also, the percentage of participants from each group who endorsed *yes* for the intention and recommendation items were compared using the chi-square test. All the data were analyzed using the Statistical Package for the Social Sciences Statistics version 22 (IBM Corp).

## Results

### Sociodemographics of the Sample

Tables 1 and 2 summarize sociodemographics for continuous and categorical variables, respectively. The mean age of all participants was 51.60 years (SD 9.55). On average, they had lived in the United States for 18.43 years (SD 10.80), and their mean age at the time of immigration to the United States was 33.5 years (SD 8.76). In terms of educational background, participants had received an average of 15.14 years of education (SD 3.27), and 72.5% (87/120) of participants reported completion of college or university or beyond. With regard to employment and income, about half of the participants (50.8%, 61/120) were currently employed, and 42.5% (51/120) reported their household monthly income including tax as US $7000 or more. Most participants (86.7%, 104/120) reported that their financial condition was fair, good, or very good. With regard to families and residences, the majority of participants were married or cohabitating (86.7%, 104/120) and were living with their spouse or children (90.0%, 108/120). The majority (90.0%, 108/120) also lived in their own or leased house or condominium. In terms of current health conditions and health-related behaviors, 36.7% (44/120) of participants reported to be in good or very good health, and 70.8% (85/120) of the participants reported to exercise at least once a week. In addition, the majority of participants were nonsmokers (95.8%, 115/120) and nondrinkers (80.8%, 97/120). More importantly, app and brochure groups were not significantly different in these baseline characteristics, as indicated by insignificant *t* test and chi-square test results.

### Experience of Breast Cancer Screening Before Intervention

Table 3 summarizes participants’ previous experiences related to three types of breast cancer screening: breast self-examination (BSE), clinical breast examination (CBE), and mammography. When asked about their awareness of the given screening exams at baseline assessment, 92.5% (111/120), 57.5% (69/120), and 77.5% (93/120) of participants responded that they had heard of the BSE, CBE, and mammography, respectively. In terms of procedure knowledge (ie, whether participants knew how the given screening exam is performed), 81.7% (98/120), 53.3% (64/120), and 75.8% (91/120) of participants reported to know the procedures of the BSE, CBE, and mammography, respectively. In addition, 78.3% (94/120), 61.7% (74/120), and 70.0% (84/120) of participants reported that they had previously performed or received the BSE, CBE, and mammogram, respectively. The rate of having performed or received each screening exam at least once every 6 months was 45.8% (55/120), 61.7% (74/120), and 70.0% (84/120) for the BSE, CBE, and mammogram, respectively. Finally, time since the last performance or receipt of each screening exam was on average 0.91 years (SD 1.83), 3.59 years (SD 4.06), and 4.30 years (SD 4.05) for the BSE, CBE, and mammography, respectively. The app and brochure groups were not significantly different in any of these previous experiences as indicated by insignificant chi-square and t test results.

**Table 1:** Sociodemographics for continuous variables by group.

Variable	App (N=60), mean (SD^a^)	Brochure (N=60), mean (SD)	All (N=120), break/>mean (SD)	Group difference	
				*t* (degrees of freedom)	*P* value
Age, in years	51.38 (8.74)	51.82 (10.36)	51.60 (9.55)	−0.25 (118)	.81
Years living in the United States	17.90 (9.65)	18.97 (11.89)	18.43 (10.80)	−0.54 (118)	.59
Age at the time of immigration to the United States	33.80 (9.78)	33.19 (7.68)	33.50 (8.76)	0.38 (118)	.71
Years of education	15.00 (2.73)	15.28 (3.75)	15.14 (3.27)	−0.47 (118)	.64

^a^SD: standard deviation.

**Table 2:** Sociodemographics for categorical variables by group.

Variable		App (N=60), n (%)	Brochure (N=60), n (%)	All (N=120), n (%)	Group difference	
					χ^2^ (degrees of freedom)	*P* value
**Highest level of education**						
	Middle school and less	4 (7)	5 (8)	9 (7.5)	2.7 (3)	.44
	Completed high school	10 (17)	14 (23)	24 (20.0)		
	Completed college or university	36 (60)	27 (45)	63 (52.5)		
	Completed graduate school	10 (17)	14 (23)	24 (20.0)		
**Employment**						
	No	28 (47)	31 (52)	59 (49.2)	0.3 (1)	.58
	Yes	32 (53)	29 (48)	61 (50.8)		
**Household monthly income in US dollars (including tax)**						
	Up to US $2999	13 (22)	15 (25)	28 (23.3)	1.3 (3)	.74
	US $3000-$6999	21 (35)	19 (32)	40 (33.3)		
	US $7000-$11,999	16 (27)	13 (22)	29 (24.2)		
	US $12,000 or more	9 (15)	13 (22)	22 (18.3)		
**Financial status**						
	Very bad	1 (2)	2 (3)	3 (2.5)	1.3 (4)	.86
	Bad	5 (8)	7 (12)	12 (10.0)		
	Fair	40 (67)	35 (58)	75 (62.5)		
	Good	13 (22)	13 (22)	26 (21.7)		
	Very good	1 (2)	2 (3)	3 (2.5)		
**Marital status**						
	Single	3 (5)	2 (3)	5 (4.2)	1.1 (3)	.78
	Married or cohabitating	51 (85)	53 (88)	104 (86.7)		
	Separated or divorced	4 (7)	2 (3)	6 (5.0)		
	Widowed	2 (3)	3 (5)	5 (4.2)		
**Family members**						
	Alone	6 (10)	6 (10)	12 (10.0)	1.1 (3)	.78
	With spouse	13 (22)	17 (28)	30 (25.0)		
	With spouse and children	36 (60)	34 (57)	70 (58.3)		
	With children (no spouse)	5 (8)	3 (5)	8 (6.7)		
**Type of residence**						
	Own house or condominium	42 (70)	37 (62)	79 (65.8)	3.6 (4)	.46
	Leased house or condominium	11 (18)	18 (30)	29 (24.2)		
	Public housing	5 (8)	2 (3)	7 (5.8)		
	Rented room	1 (2)	2 (3)	3 (2.5)		
	Others	1 (2)	1 (2)	2 (1.7)		
**Current health condition**						
	Very bad	1 (2)	0 (0)	1 (0.8)	7.7 (4)	.10
	Bad	7 (12)	9 (15)	16 (13.3)		
	Fair	29 (48)	30 (50)	59 (49.2)		
	Good	13 (22)	19 (32)	32 (26.7)		
	Very good	10 (17)	2 (3)	12 (10.0)		
**Number of exercises per week**						
	0	19 (32)	16 (27)	35 (29.2)	1.6 (4)	.81
	1-2	19 (32)	22 (37)	41 (34.2)		
	3-4	15 (25)	18 (30)	33 (27.5)		
	5-6	5 (8)	3 (5)	8 (6.7)		
	7+	2 (3)	1 (2)	3 (2.5)		
**Smoking**						
	No	57 (95)	58 (97)	115 (95.8)	.^a^ (1)	>.99
	Yes	3 (5)	2 (3)	5 (4.2)		
**Drinking**						
	No	51 (85)	46 (77)	97 (80.8)	1.4 (1)	.25
	Yes	9 (15)	14 (23)	23 (19.2)		

^a^Dot signifies that no numeric value is available. Instead of Pearson chi-square test, Fisher exact test was performed, given that the expected count for some cells is less than 5. In the Statistical Package for the Social Sciences (SPSS) software, only the *P* value of the Fisher exact test is reported rather than the test statistic.

**Table 3:** Summary of previous experience of breast cancer screening by group.

Screening			App (N=60), n (%)	Brochure (N=60), n (%)	All (N=120), n (%)	Group difference	
						χ^2^ (degrees of freedom)	*P* value
**BSE**^a^							
	**Awareness**						
		Yes	54 (90)	57 (95)	111 (92.5)	.^b^ (1)	.49
		No	6 (10)	3 (5)	9 (7.5)		
	**Procedure knowledge**						
		Yes	48 (80)	50 (83)	98 (81.7)	0.2 (1)	.64
		No	12 (20)	10 (17)	22 (18.3)		
	**Previous performance**						
		Yes	49 (82)	45 (75)	94 (78.3)	0.8 (1)	.38
		No	11 (18)	15 (25)	26 (21.7)		
	Years since the last performance, mean (SD^c^)		1.04 (2.24)	0.78 (1.28)	0.91 (1.83)	−1.15 (90)^d^	.25
**CBE**^e^							
	**Awareness**						
		Yes	34 (57)	35 (58)	69 (57.5)	0.0 (1)	.85
		No	26 (43)	25 (42)	51 (42.5)		
	**Procedure knowledge**						
		Yes	29 (48)	35 (58)	64 (53.3)	1.2 (1)	.27
		No	31 (52)	25 (42)	56 (46.7)		
	**Previous receipt**						
		Yes	37 (62)	37 (62)	74 (61.7)	0.0 (1)	>.99
		No	23 (38)	23 (38)	46 (38.3)		
	Years since the last receipt, mean (SD)		4.22 (4.22)	2.97 (3.86)	3.59 (4.06)	−1.51 (72)^d^	.13
**Mammography**							
	**Awareness**						
		Yes	47 (78)	46 (77)	93 (77.5)	0.1 (1)	.83
		No	13 (22)	14 (23)	27 (22.5)		
	**Procedure knowledge**						
		Yes	45 (75)	46 (77)	91 (75.8)	0.1 (1)	.83
		No	15 (25)	14 (23)	29 (24.2)		
	**Previous receipt**						
		Yes	44 (73)	40 (67)	84 (70.0)	0.6 (1)	.43
		No	16 (27)	20 (33)	36 (30.0)		
	Years since the last receipt, mean (SD)		4.23 (3.15)	4.38 (4.89)	4.30 (4.05)	−0.02 (82)^d^	.98

^a^BSE: breast self-examination.

^b^Dot signifies that no numeric value is available. Instead of Pearson chi-square test, Fisher exact test was performed, given that the expected count for some cells is less than 5. In the Statistical Package for the Social Sciences (SPSS) software, only the *P* value of the Fisher exact test is reported rather than the test statistic.

^c^SD: standard deviation.

^d^Signifies *t* (degrees of freedom).

^e^CBE: clinical breast examination.

### Change in Knowledge, Attitudes, and Beliefs About Breast Cancer and Screening After the Intervention (Hypothesis 1)

**Figure 1:**
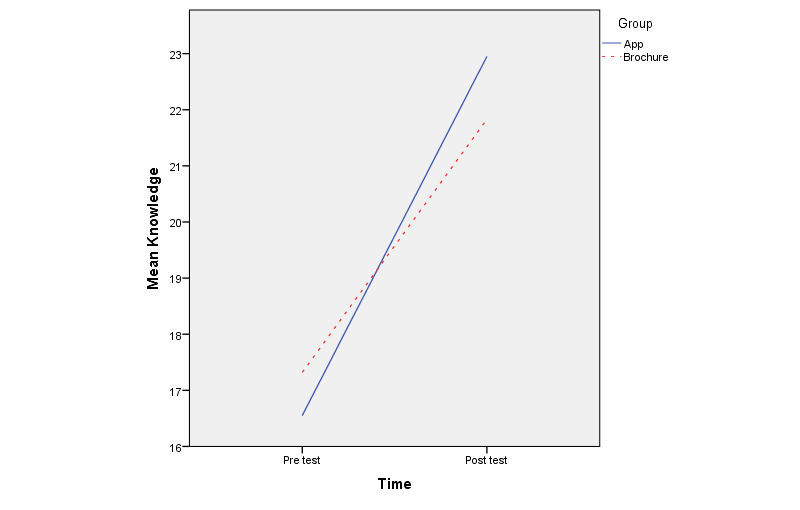
Interaction between time and group.

Table 4 summarizes the changes in knowledge, attitudes, and beliefs about breast cancer screening by group as well as results of mixed-design ANOVA *.* Independent-samples *t* test results were insignificant at the pretest, indicating that the two groups were not significantly different in any of these constructs at baseline. Demonstrating within-subjects effects, participants showed significant improvement in knowledge (*F*_1,118_=209.74, *P*<.001, effect size=0.64), reduction in fatalism (*F*_1,118_=15.19 , *P*<.001, effect size=0.11), increased perceived benefits (*F*_1,118_=20.16 , *P*<.001, effect size=0.15), and increased self-efficacy (*F*_1,118_=18.79 , *P*<.001, effect size=0.14) related to breast cancer and screening. However, none of the between-subjects effects were significant, indicating that for both the pre- and posttests, the participants’ scores on knowledge, attitudes, and beliefs about breast cancer and screening were not substantially different between the app and brochure groups. With regard to interaction between time and group, only the knowledge construct was found to be significant (*F*_1,118_=6.24, *P*=.01, effect size=0.05) indicating that the increase in knowledge between pre- and posttest was significantly larger for the app group compared to the brochure group. The nature of this interaction is displayed in Figure 1.

**Table 4:** Summary of change in knowledge, attitude, and belief about breast cancer screening by group.

Variables	App (N=60)			Brochure (N=60)			Group difference		All (N=120)			Mixed-design ANOVA^a^		
	Pretest, mean (SD^b^)	Posttest, mean (SD)	Pretest, mean (SD)		Posttest, mean (SD)	*t* (degrees of freedom)		Pretest, mean (SD)		Posttest, mean (SD)	Within-group, *F*^c^ (effect size^d^)		Between- group, *F* (effect size)	Interaction time × group, *F* (effect size)
Knowledge on breast cancer and screening	16.55 (5.13)	22.95 (3.52)	17.32 (4.38)		21.83 (3.48)	−0.88 (118)		16.93 (4.77)		22.39 (3.53)	209.74^e^ (0.64)		0.069 (0.00)	6.24^f^ (0.05)
Barriers to receiving mammography	27.22 (8.69)	26.10 (7.27)	26.60 (6.43)		25.88 (6.63)	0.44 (118)		26.91 (7.62)		25.99 (6.93)	2.66 (0.02)		0.12 (0.00)	0.13 (0.00)
Distrust of health professionals	10.10 (2.47)	9.57 (1.82)	10.23 (2.27)		9.98 (1.94)	−0.31 (118)		10.17 (2.36)		9.78 (1.88)	3.66 (0.03)		0.68 (0.01)	0.48 (0.00)
Fatalism	6.28 (1.54)	5.52 (1.24)	5.88 (1.56)		5.48 (1.61)	1.41 (118)		6.08 (1.56)		5.50 (1.43)	15.19^e^ (0.11)		0.90 (0.01)	1.50 (0.01)
Fear of discovery	1.55 (0.67)	1.65 (0.66)	1.52 (0.62)		1.52 (0.57)	0.28 (118)		1.53 (0.65)		1.58 (0.62)	0.78 (0.01)		0.69 (0.01)	0.78 (0.01)
Modesty	10.82 (2.72)	11.05 (2.56)	10.87 (2.71)		10.63 (2.23)	−0.10 (118)		10.84 (2.70)		10.84 (2.40)	0.00 (0.00)		0.20 (0.00)	1.09 (0.01)
Perceived benefits	15.12 (2.85)	16.42 (2.12)	15.07 (1.89)		15.82 (2.21)	0.11 (118)		15.09 (2.40)		16.12 (2.18)	20. 16^e^ (0.15)		0.86 (0.01)	1.45 (0.01)
Perceived susceptibility	5.62 (2.03)	5.43 (1.37)	5.32 (1.14)		5.35 (1.12)	1.00 (118)		5.47 (1.64)		5.39 (1.25)	0.27 (0.00)		0.73 (0.01)	0.56 (0.01)
Prevention orientation	15.77 (1.97)	16.00 (2.17)	15.98 (1.93)		15.93 (2.15)	−0.61 (118)		15.88 (1.94)		15.97 (2.15)	0.24 (0.00)		0.05 (0.00)	0.57 (0.01)
Self-efficacy on breast cancer screening	23.82 (4.54)	25.20 (4.27)	22.98 (3.98)		24.53 (4.06)	1.07 (118)		23.40 (4.27)		24.87 (4.16)	18.79^e^ (0.14)		1.18 (0.01)	0.06 (0.00)
Social support	15.77 (2.94)	16.23 (3.03)	15.65 (2.85)		15.98 (2.70)	0.22 (118)		15.71 (2.89)		16.11 (2.86)	3.07 (0.03)		0.15 (0.00)	0.09 (0.00)

^a^ANOVA: analysis of variance.

^b^SD: standard deviation.

^c^Degrees of freedom for *F* test=1,118.

^d^For the effect size, partial eta squared (η_p_^2^) was computed.

^e^*P*<.001.

^f^*P*<.05.

**Table 5:** Intention for mammography use by group.

Response	App (N=60)		Brochure (N=60)		All (N=120)		Group difference	
	Pretest, n (%)	Posttest, n (%)	Pretest, n (%)	Posttest, n (%)	Pretest, n (%)	Posttest, n (%)	Pretest, *t* (degrees of freedom)	Posttest, *t* (degrees of freedom)
No plan to do within 1 year	8 (13)	9 (15)	10 (17)	10 (17)	18 (15.0)	19 (15.8)	−0.64 (118)	3.48^a^ (118)
Plan to do within 1 year	8 (13)	24 (40)	8 (13)	40 (67)	16 (13.3)	64 (53.3)		
Plan to do within the next 3 months	0 (0)	12 (20)	1 (2)	7 (12)	1 (0.8)	19 (15.8)		
Plan to do within 1 month	0 (0)	14 (23)	1 (2)	1 (2)	1 (0.8)	15 (12.5)		

^a^*P*<.01.

### Change in Intention to Receive Breast Cancer Screening After the Intervention (Hypothesis 2)

Table 5 summarizes intention for mammography use by group. Independent-samples *t* test results were insignificant at the pretest, indicating that intention for mammography use was not substantially different between groups at the pretest (*t*_118_=−0.64, *P*=.53). Therefore, instead of mixed-design ANOVA *,* an independent-samples *t* test was performed solely for the posttest scores to examine group differences in intention for mammography use after the intervention. As shown in Table 5, a significant group difference was found (*t*_118_=3.48, *P*=.001) with a combined sample size of 120.

### Receipt of Mammography After the Intervention (Hypothesis 3)

The app group was found to receive mammograms significantly more than the brochure group after the intervention, as indicated by chi-square test results (χ^2^_1_=24.4, *P*<.001). Specifically, 75% (45/60) of app group participants versus 30% (18/60) of brochure group participants received a mammogram after the intervention. About 8% (5/60) of participants in the app group received a mammogram through health navigators who arranged appointments and provided transportation and interpretation services, whereas 33% (20/60) received a mammogram at the Mammo a-go-go program, a free mammogram event that health navigator arranged in conjunction with a local health care system for participants who did not have health insurance or were underinsured. The rest of the participants (20/60, 33%) received a mammogram by themselves without a health navigator’s help.

**Table 6:** Satisfaction, effectiveness, and acceptability of the intervention by group.

Variable		App (N=60)	Brochure (N=60)	All (N=120)	Group difference
		n (%)	n (%)	n (%)	*t* (*P* value)
**Effectiveness**					
	Very ineffectual	2 (3)	1 (2)	3 (2.5)	3.73 (<.001)
	Ineffectual	0 (0)	2 (3)	2 (1.7)	
	Effectual	26 (43)	49 (82)	75 (62.5)	
	Very Effectual	32 (53)	8 (13)	40 (33.3)	
**Increase of knowledge**					
	Same	0 (0)	4 (7)	4 (3.3)	3.52 (.001)
	Improved	38 (63)	48 (80)	86 (71.7)	
	Very improved	22 (37)	8 (13)	30 (25.0)	
**Satisfaction with intervention**					
	Very dissatisfied	0 (0)	0 (0)	0 (0.0)	3.03 (.003)
	Dissatisfied	0 (0)	1 (2)	1 (0.8)	
	Satisfied	36 (60)	49 (82)	85 (70.8)	
	Very satisfied	24 (40)	10 (17)	34 (28.3)	
**Intention to receive a mammography** **in the future**					
	Yes	57 (95)	54 (90)	111 (92.5)	.^a^ (.49)
	No	3 (5)	6 (10)	9 (7.5)	
**Recommendation of mammography**					
	Yes	59 (98)	55 (92)	114 (95.0)	.^a^ (.21)
	No	1 (2)	5 (8)	6 (5.0)	

^a^Dot signifies that no numeric value is available. Instead of Pearson chi-square test, Fisher exact test was performed given that the expected count for some cells is less than 5. In Statistical Package for the Social Sciences software, only the *P* value of the Fisher exact test is reported rather than the test statistic.

### Satisfaction With and Effectiveness of the Intervention (Hypothesis 4)

To examine group differences in satisfaction with and effectiveness of the intervention, independent-samples *t* test and chi-square test were performed for Likert-type items and dichotomous items, respectively. As shown in Table 6, compared with the brochure group, the app group reported significantly higher ratings on perceived effectiveness of the intervention (*t*_118_=3.73, *P*<.001), increase in knowledge (*t*_118_=3.52, *P*=.001), and satisfaction with the intervention (*t*_118_=3.03, *P*=.003). Although the app group also expressed greater intention to receive a mammogram in the future when it is due (95%, 57/60 vs 90%, 54/60) and were more willing to recommend the intervention they received to their friends (98%, 59/60 vs 92%, 55/60) compared with the brochure group, these differences were not statistically significant.

## Discussion

### Principal Findings

This pilot study offers initial evidence for the feasibility and effectiveness of a mobile app intervention with health navigation services as compared with the control group to increase participation in mammography among Korean American women, a hard-to-reach community with low rates of breast cancer screening. The main finding that the intervention group received mammograms at a significantly higher rate than the control group highlights breast cancer screening as another area in which innovative mobile phone app interventions can positively influence health behaviors. As such, this study further diversifies the list of outcomes shown to be compatible with an mHealth approach, contributing to a list that already includes fatigue [62], diabetes management [63], blood pressure control [64], and physical activity [65], among others [66].

Part of the differential effect of the intervention may be explained by the intervention group’s higher ratings of perceived effectiveness and satisfaction with the mobile app with health navigation services compared with the control group’s perspectives regarding the brochure. The substantial positive association between participants’ perceptions of effectiveness and the actual effect of the intervention on attitudes and behaviors has been previously established through meta-analysis, with evidence that perceived effectiveness stands as a causal influence for actual effectiveness [67,68]. There is also strong evidence that patient satisfaction with care (*care* represented in this case by the mobile app intervention) impacts clinical outcomes, including adherence to recommended behavior regimens and use of preventive care services [69]. Beyond greater perceived effectiveness and satisfaction with the intervention, the mobile app group exhibited greater increases in their knowledge levels about breast cancer screening than the control group, suggesting superior effectiveness of the mobile app for education on this topic. The link between knowledge of risk factors and screening procedures and receipt of breast cancer screening has been well established [70-72]. In addition, this finding supports the proliferation of mobile phone apps for the delivery of cancer-related information, including the effort to ensure the inclusion of scientifically validated data [73-75].

Despite an overall promising result, several of the secondary outcomes demonstrated a lack of significant differences between the intervention and control groups, contrary to hypotheses. Both the participants who received the mobile app intervention with health navigation services and those who received the usual care brochure demonstrated gains in attitudes toward screening and beliefs about barriers, self-efficacy, and health professionals over the study period; however, these gains were roughly equivalent between groups. Similarly, the percentage of women in each group who intended to receive a mammogram approximately doubled following the intervention period; however, the intervention group showed significantly higher readiness for mammography. Potential contributors to the lack of substantial differences in these outcomes include the influence of social desirability and potential contamination or spillover effects. Not only has research shown that women tend to score higher on measures of social desirability bias [76-78] but that participants from Asian cultures may score higher than groups of European descent as well [79,80]. Participants in both groups may have felt pressure to respond in positive ways on posttest measures. In addition, because participants were recruited from a local community and may have interacted outside the study context, participants in the control condition may have indirectly been exposed to contents from the intervention. The difficulty of preventing contamination has been cited as a unique barrier to conducting RCTs among Asian American populations in cancer screening research [81].

### Limitations

The interpretation of findings from this study should take certain limitations into consideration. First, some of the construct measures had low reliability, as demonstrated by coefficient alphas below .7 for prevention orientation, fatalism, and social support. Because unreliable scales decrease the statistical power of instruments, the low reliability in these measures may have contributed to the insignificant differences found between the intervention and control groups. Second, there are multiple potential confounding factors that influenced mammogram receipt in both groups, including the monthly phone calls to check receipt of mammogram over the 6-month follow-up period, a sense of obligation that participants may have felt based on the rapport developed with the research team, and the pressure to comply with expectations based on a Korean cultural norm that makes women reluctant to give a direct negative response to a request. Third, the provision of health navigator services (eg, providing interpretation services and transportation services and arranging a free mammogram event such as Mammo-a-go-go program) to the intervention group may have been responsible for part of the differential effect of the intervention on the primary and secondary outcomes. However, for an immigrant group that lacks English proficiency and health care accessibility, provision of health navigation services is critical, combined with mobile app program to promote mammography. This study design renders it impossible to parse out the additive effect of these factors. Future studies, therefore, should use a three-arm design (app vs app plus health navigation services vs usual care) to tease out the pure effectiveness of mobile app intervention as compared with the mobile app intervention with health navigation services and usual care.

### Implications for Practice, Policy, and Future Research

Highlights from postintervention focus groups shed light on ways that the information garnered from this research can more broadly enhance cancer prevention efforts that rely on mobile technology as an intervention medium. Overall, participants in the intervention group provided feedback that the mobile app helped to increase their knowledge about breast cancer and screening methods, reminding them of the importance of receiving regular mammograms. Interestingly, their participation in the study also primed them to be more attentive to information regarding breast cancer when it incidentally arose during their consumption of other media such as television and radio. This feedback highlights the potential for mobile phone messaging to act as a conduit of other sources of cancer-related information and screening motivation. Participants’ comments also suggest that simply increasing knowledge of breast cancer risk factors may induce lifestyle changes to promote cancer prevention, such as regular exercise and diet. In addition, incorporating media into direct education about the procedures for screening methods, such as a breast self-exam video and a detailed procedural video for mammography embedded in the mobile app, may promote greater engagement in self-screening methods and efficiently alleviate fears toward screening. Health care clinics may capitalize on this information by showing such videos during patient visits or sending out intermittent educational and interactive multimedia messages during the interim between visits. Finally, the culturally unique aspects of the app, such as having information available in participants’ native language and having testimonials from peers who share the same ethnic background, appear to have been particularly important components, reinforcing the notion that tailoring is essential in mHealth research and outreach efforts.

On the basis of the ubiquity of mobile phones in society, including widespread use among minority communities, a multilevel and multimedia messaging intervention such as mMammogram combined with health navigator services and locally available free mammogram program (eg, Mammo-a-go-go program) holds promise to be an effective method in reaching hard-to-recruit populations with high breast cancer burdens. The use of tailored digital messages that cover broad content areas overcomes restrictions based on place and time of delivery, as well as resource and financial limits. The format and contents of mMammogram could be easily translated and disseminated to various ethnic groups who face barriers to cancer screening, with each iteration of the model programmed to tailor its approach to the unique needs of the cultural group and the individual. Along the same lines, the mMammogram model could be modified to target promotion of multiple different preventive screening behaviors to protect against other cancers such as colorectal cancer. In addition, apps could be designed to cover the full spectrum of cancer prevention, treatment, and survivorship, encompassing diagnosis, treatment options, decision making, communication strategies, psychosocial care, and wellness. By refining and expanding technologies that target disadvantaged populations, mHealth initiatives offer an encouraging strategy to reduce disparities in breast cancer and other health conditions.

## Abbreviations

ACS: American Cancer Society
ANOVA: analysis of variance
BSE: breast self-examination
CAB: community advisory board
CBE: clinical breast examination
FBM: Fogg behavioral model
GPS: global positioning system
HBM: health belief model
mHealth: mobile health
RCT: randomized controlled trial
SD: standard deviation

## Appendix

Multimedia Appendix 1 CONSORT‐EHEALTH checklist (V.1.6.1).
